# Lung-Directed Bacteriotherapy in Cystic Fibrosis: Could It Be an Option?

**DOI:** 10.3390/antibiotics11030326

**Published:** 2022-02-28

**Authors:** Giovanna Batoni, Giuseppantonio Maisetta, Esingül Kaya, Semih Esin

**Affiliations:** Department of Translational Research and New Technologies in Medicine and Surgery, University of Pisa, Via S. Zeno 37, 56123 Pisa, Italy; giuseppantonio.maisetta@unipi.it (G.M.); e.kaya@studenti.unipi.it (E.K.); semih.esin@unipi.it (S.E.)

**Keywords:** cystic fibrosis, probiotics, *Lactobacillus*, bacteriotherapy, aerosol administration, gut–lung axis

## Abstract

Due to the alarming spread of bacterial resistance to conventional drugs, the sole use of antibiotics to fight lung infections in cystic fibrosis (CF) is not resolutive, and novel strategies to replace or complement the use of antibiotics are highly desirable. Among these strategies, the use of probiotics is emerging as a particularly attractive approach. Probiotic administration via the oral route has demonstrated an ability to improve lung function and to reduce infection and exacerbation rates in CF patients through mechanisms mainly attributable to the gut–lung axis. Nevertheless, some studies reported no beneficial effect of probiotic intake suggesting that there is margin for improvement of such innovative intervention in CF. The present review aims to address the rationale behind probiotic use in CF and discuss the hypothesis that nasal/aerosol administration of appropriate probiotic strains may help to exert a direct beneficial effect on the respiratory tract, increasing the effectiveness of probiotic interventions in CF patients.

## 1. Introduction

**Figure 1:**
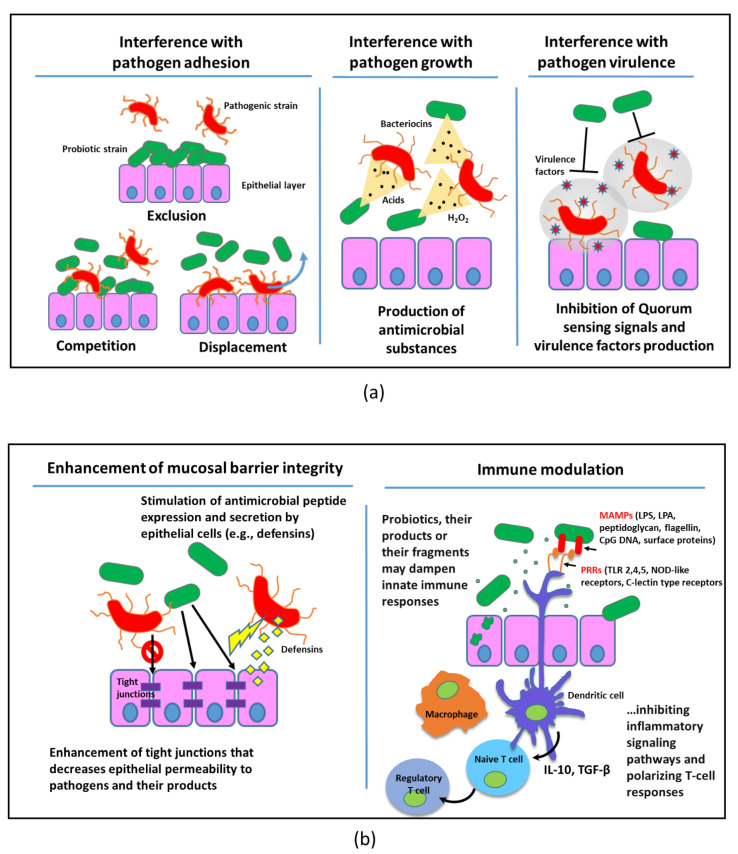
Mechanisms of the antimicrobial effects of probiotics at the mucosal interfaces: (**a**) direct effects; (**b**) indirect effects.

In the era of antibiotic resistance, the search for new therapeutic strategies as alternatives or adjuvants of antibiotic use is a widely recognized priority. One of these strategies, referred to as bacteriotherapy, relies on the use of harmless bacteria to compete with pathogenic bacteria with the aim of contrasting the growth of the pathogens and/or the expression of their virulence traits [1,2]. The interest in the use of bacteriotherapy to prevent or cure human diseases has been markedly boosted by the advent of high throughput molecular analyses such as next generation sequencing and “omics” technologies that have revealed that each body site has its own health-associated microbiota and that disturbance of this microbiota (i.e., dysbiosis) might be associated with a number of infectious and non-infectious human disorders. The main tool of bacteriotherapy is the administration of probiotics defined by the Food and Agriculture Organization of the United Nations and by the World Health Organization (FAO/WHO) as live organisms that when administered in adequate amounts confer health benefits to the host [3]. The mechanisms of action of probiotics are numerous and include competition with pathogenic microorganisms for adhesion sites, production of metabolites that antagonize pathogen growth and/or inhibit virulence factor production, dampening of inflammation and modulation of the host’s immune response (Figure 1).

Most probiotics currently in use are Gram-positive bacteria belonging to the *Lactobacillus* and *Bifidobacterium* genera, but the list of genera/species with potential beneficial effects is rapidly expanding and next generation of probiotics are being evaluated for future use [4]. So far, some of the main fields of probiotics applications are the prevention/treatment of intestinal disorders (e.g., acute and antibiotic-induced diarrhea, relapsing gastroenteritis due to *Clostridioides difficile*, chronic inflammatory diseases of the digestive tract) or the correction of dysbiosis of the female uro-genital tract (e.g., bacterial vaginosis), but many other medical indications are being investigated, including the prevention and treatment of Coronavirus Disease 2019 (COVID-19) [2,5,6,7,8]. Among these, the impact of probiotics on respiratory tract infections has gained growing interest in the last few years based on the awareness that the lung has its own microbiota and that lung dysbiosis is associated with a number of both acute and chronic pathologic conditions, including cystic fibrosis (CF) [9].

CF is a systemic disease caused by genetic mutations of the CF Transmembrane Conductance Regulator (CFTR), a chloride channel expressed by epithelial cells, involved in ion and fluid homeostasis [10]. Lack/dysfunction of this regulator leads to the production of a thick, sticky mucus in the respiratory, digestive and reproductive systems, as well as an increased salt concentration in sweat. Respiratory complications include a persistent cough, bronchiectasis, sinusitis and repeated lung infections due to mucus stagnation and altered clearance that favor bacteria persistence [10]. This condition, in turn, causes influx of immune cells and chronic inflammation with consequent tissue damage and lung function decline [10]. Recurrent pulmonary infections are the major cause of the morbidity and mortality of CF patients, and often require frequent hospitalizations and prolonged administration of antibiotics. During the course of the disease, the diversity of the lung microbiome progressively decreases and one or more pathogenic species become prevalent, mainly including *Staphylococcus aureus*, *Pseudomonas aeruginosa*, *Burkholderia cepacea* complex [11]. Many of these species are strong biofilm producers and switching to the biofilm mode of growth not only promotes their persistence in the lung, but also protects them from the action of antibiotics. In addition, the continuous exposure of patients to antibiotics in the attempt to control the infections contributes to the insurgence of multidrug-resistant pathogens supporting the loss of microbiome diversity with age due to antibiotics use, with a parallel increase in disease severity [12].

In this scenario, the potential use of probiotic bacteria, either as prophylactic agents for preventing or delaying pulmonary colonization with CF pathogens, or as therapeutic tools to fight established pulmonary infections in CF has attracted considerable interest in recent years [13,14]. The advantage of using probiotics in CF could be twofold: from one side, they could contribute directly or indirectly to pathogens’ elimination (Figure 1); from the other side, they could help in the reconstitution/maintenance of a health-associated microbiome [15], possibly reducing re-infection rates and/or exacerbations. 

The aim of the present review is to highlight the rationale behind the use of probiotics in CF, and to discuss the hypothesis that a lung-directed bacteriotherapy, through airway administration of probiotics, could represent an alternative to probiotic diet supplementation to improve the benefits and clinical outcomes of this kind of intervention in CF. 

## 2. Oral Administration of Probiotics as a Therapeutic Strategy in CF

Several lines of evidence support the administration of probiotics via the oral route in CF patients, although this measure still requires a full demonstration of its clinical efficacy [13,14] (Table 1).

**Figure 2:**
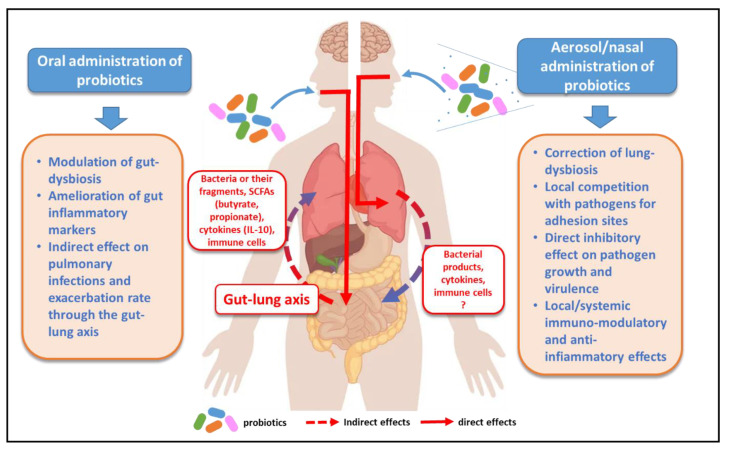
Oral versus aerosol probiotic administration in cystic fibrosis. SCFAs: short chain fatty acids. Although the gut–lung axis is considered bidirectional, the influence of lung microbiota on intestinal immunity has been poorly explored so far.

Firstly, qualitative and quantitative changes in the intestinal microbiota of CF patients have been widely documented, suggesting that probiotic supplementation through dietary intake as food products may help to correct the dysbiosis and restore a health-associated gut microbiota [18]. Secondly, evidence is rapidly accumulating that gut microbiota not only modulate the environment and host immunity locally but are also able to influence distant organs such as the lung, due to the existence of bidirectional communications between the gut and lungs called the gut–lung axis [30,31,32]. Thus, modulation of the dysbiotic microbiota in CF patients through probiotic intake could also exert a beneficial indirect effect on lung-function, possibly by reducing the rate of infections and/or exacerbation (Figure 2). The following two paragraphs will address these issues in more detail. 

### 2.1. Probiotic Supplementation to Correct Gut Dysbiosis in CF

In a healthy status, the human gut microbiota are highly diverse, harboring a total of 395 bacterial phylotypes with *Firmicutes* and *Bacteroidetes* being the dominant phyla [33]. It establishes early in life and performs a number of protective roles considered essential in ensuring the normal functioning of the human host [34]. These include: (i) the barrier effect that prevents invasion by pathogenic microorganisms; (ii) the production of metabolites with a wide variety of biological effects (e.g., vitamins); (iii) the fermentation of complex, non-digestible dietary carbohydrates with production of short chain fatty acids (SCFAs) with anti-inflammatory and anti-oxidant potential; (iv) the stimulation and modulation of the host immune system, as demonstrated by the extensive deficits in immune functions exhibited by germ-free animals [34,35,36].

Several studies have reported alterations of the intestinal microbiota in CF patients [31,37]. Such alterations might be due to several factors [32]. The intensive antibiotic administration to treat chronic lung infections not only has a deleterious effect on the lung-microbiome of CF patients, but also affects the diversity and metabolic functioning of the gastrointestinal microbiota, potentially leading to a state of dysbiosis. Furthermore, the high-fat diet often followed by CF patients due to absorption defects and difficulty in weight-gain is known to have a negative impact on microbiome diversity and abundance [30]. Finally, due to the CTFR defect in the intestine, bile acid production and secretion of pancreatic enzymes and bicarbonate are altered, leading to a more acidic intestinal pH and to a higher fecal fat content, all of which have possible effects on shaping the gut microbiota [31,32]. 

Although the relative frequency of specific taxa may vary in different studies depending on the cohort of patients examined (age, geographic location, severity of the disease, etc.), it is generally recognized that the stool microbiota of CF patients exhibit a decreased diversity as compared to healthy controls [31]. For instance, Duytschaever and coworkers analyzed the predominant fecal microbiota composition of a group of pediatric patients with CF as compared to their healthy siblings [37]. By using cross-sectional and longitudinal approaches, they demonstrated a reduction in the *Bacteroides* and *Prevotella* count and pronounced differences in species richness between CF patients and sibling samples. Other studies have documented that within the *Firmicutes*, CF patients show a decrease in beneficial, SCFA-producing microorganisms at the genus level (i.e., *Faecalibacterium*, *Roseburia*, *Akkermansia*, *Eubacterium*, *Alistipes* and *Odoribacter*) and an increase in potentially pathogenic and SCFA-degrading microorganisms, such as *Enterococcus spp.* or *Clostridioides difficile* [31]. Some changes seem to be age-related such as the relative increase in *Proteobacteria* (*Escherichia*, *Shigella*, *Enterobacter* and *Morganella*), mainly reported in studies focusing on CF children and adolescents [38,39]. Interestingly, genera often involved in lung infections, such as *Staphylococcus* and *Streptococcus*, are also augmented in fecal samples [40,41]. Microbiota alteration is not only at the taxonomic level, but also implies a functional dysbiosis as demonstrated in a recent study reporting an enrichment of genes involved in SCFA, antioxidant and nutrient metabolism, all of which are relevant to growth and nutrition in CF [38]. 

The recent advent of the triple CTFR modulator therapy has drastically revolutionized the management of CF patients and has represented a fundamental step forward in halting disease progression and improving the quality of life of most CF patients [42]. Nevertheless, still few studies address the effects of CTFR-targeted modulator therapies on gut microbiota. Although the new therapies seem to improve intestinal functions (e.g., normalization of intestinal pH, enhancement of pancreatic enzyme function, improved intestinal nutrient absorption) their effects on gut microbiota dysfunction seem minimal [43,44,45].

Given all these premises, restoring the balance of intestinal microbiota in CF patients by introducing probiotics via the oral route seems a promising approach. In this context, Bruzzese et al. implemented a double-blind clinical trial to evaluate the effects of *Lactobacillus rhamnosus* GG (LGG) administration to children with CF on intestinal microflora composition and inflammation [18]. They reported that administration of 6 x 10^9^ CFU of LGG once daily for 1 month partially restored the composition of intestinal microbiota, causing a statistically significant increase in *Bacteroidetes* counts as compared to children treated with placebo. In agreement with a previous trial, they also observed a parallel reduction in the levels of intestinal inflammation markers such as fecal calprotectin and rectal nitric oxide [17,18]. In another study, *Lactobacillus reuteri* DSM 17,938 was administered to CF patients (age range 8–44 years) for 6 months as probiotic preparation containing 10^8^ CFU in a chewable tablet (one tablet per day) [21]. Total fecal DNA was obtained and metagenomic pyrosequencing analysis was carried out to assess the microbiome composition. Marked dysbiosis was observed in the study group at the baseline with a high concentration of *Proteobacteria*. Following *L. reuteri* intake, a reduction in the total bacterial density was observed, mostly due to a considerable reduction in the γ-*Proteobacteria* phylum; a tendency to increase microbial diversity was also seen with a higher representation of *Firmicutes*. Importantly, patients reported no adverse effects during probiotic administration and an overall improvement of the gastrointestinal comfort evaluated via the Gastrointestinal Quality of Life Index (GIQLI).

### 2.2. Probiotic Supplementation to Correct Lung Dysbiosis in CF through the Gut–Lung Axis

In addition to locally directed effects on intestine ecology, evidence is progressively accumulating that probiotic supplementation in CF may exert indirect effects at the lung level through the gut–lung axis [22,23] (Figure 2). The molecular mechanisms involved in these processes are complex and still far from being fully elucidated, but they surely represent a fascinating area of research and a possible source of therapeutic interventions in CF. The influence of gut microbiota on lung functions is thought to be mainly mediated by the immunological crosstalk that establishes between the gut and the lung through the blood and lymphatic circulation. Nevertheless, there are also suggestions of a direct transmission of metabolites and/or microbes from the gut to the airway [31,32]. 

The ability of probiotic strains to modulate the local and systemic immune response is linked to the expression of different microorganism-associated molecular patterns (MAMPs) (e.g., lipopolysaccharides, lipoteichoic acid, peptidoglycan, flagellin, CpG-DNA, surface (lipo)proteins) that are recognized by pattern recognition receptors (PRRs) on host epithelial and innate immune cells (e.g., Toll-like receptors, NOD-like receptors, C-lectin type receptors) [46] (Figure 1b). Interaction between MAMPs and PRRs leads to the activation of multiple signaling cascades with the activation of the NF-kB factor and the expression of many genes regulating innate immunity and inflammation that generates a molecular response against the encountered microorganisms. Cytokines, chemokines and other host immune modulators (e.g., SCFAs, vitamins) may enter the blood circulation also favored by the high intestinal permeability of CF patients [47,48], and exert their effects in distant organs such as the lung. Notably, the array of MAMPs expressed by probiotics is strain-specific, suggesting that different probiotic strains may evocate differential immunological responses [49,50]. 

The systemic probiotic effect might also be mediated by dendritic cells (DC), considered essential players in linking innate and adaptive immunity. Probiotics interact with and are internalized by DC localized between intestinal cells or in the submucosal region [46]. Probiotic-loaded DC travel to the draining lymph nodes where they promote the activation of various T-cell subsets (including T reg cells) and the production of regulatory cytokines such as IL-10, TGF-β, IFN-γ and IL-6 [46,51]. In lymph nodes, activated T cells gain the capacity to home in on sites of infection or antigen exposure through the acquisition of homing molecules thereby exercising their effector functions in extra-intestinal organs [51].

Microbial metabolites may also play an important role in the gut–lung axis. Among them, particular importance is attributed to SCFAs, products of the microbial fermentation of dietary fibers that include acetate, butyrate and propionate. In particular, butyrate seems to exert broad anti-inflammatory activities through the suppression of lipopolysaccharide (LPS)-induced NF-κB activation and stimulation of the production of the anti-inflammatory cytokine IL-10 by human monocytes [52]. 

Finally, suggestions exist that a direct microbial translocation may occur between the gut and the lung [31,53]. For example, the gut and respiratory microbiome in CF infants displays a certain degree of overlapping dominated by *Veillonella* and *Streptococcus* [54]. Furthermore, a serial analysis of the gut and respiratory microbiome in CF infants has demonstrated that a significant proportion of genera (14/16) that underwent a significant increment in the gut also experienced an increment in the respiratory tract. For 7 genera, even gut colonization preceded their appearance in the respiratory tract [54]. Such observations have important implications as they suggest that specific dietary or probiotic interventions may be an effective means to change the course of the colonization of the CF lung, thereby improving patient outcomes.

It is important to note that the gut–lung influence might be bidirectional. That is, the gut microbiota influences lung health outcomes, but a communication in the opposite direction is also likely to occur with the lung influencing the structure of gut microbiota and the intestinal inflammatory status (Figure 2).

### 2.3. Clinical Studies of Probiotic Supplementation in CF

Several clinical studies have attempted to establish whether diet supplementation of probiotics has a positive effect on lung function and rate of infections and exacerbations in CF. A detailed review of such studies is out of the scope of this article as several recent meta-analyses have widely addressed this specific issue [14,26,27,28,55]. Although most of the studies are concordant in reporting reduction in respiratory exacerbations in CF patients and improvement of respiratory function following probiotic intake [22,23,26,27,28,29,56,57], discrepant results of poor or no effect of probiotics supplementation in CF also exist [24,25], leaving the question of the effectiveness of such interventions in CF open. 

Several factors may explain the discordant results reported in different studies including dosage, formulation, administration route, length of the therapeutic intervention, probiotic strain(s) administered, as well as age, severity of the disease and type of CTFR mutation of the study groups. In addition, an overall methodological limitation in the design of clinical studies addressing the beneficial effects of probiotic supplementation has been recently highlighted, [26,27,28] posing doubts on the generalization of the results obtained in some studies. All these factors do not permit, at the moment, an evidence-based recommendation of probiotic use in CF patients, but the subject holds expectations and hopes. Well-designed in vitro, ex vivo and large-scale clinical studies are needed to further corroborate the safety and efficacy of probiotics and to identify the specific probiotic strains, dose, and timing of administration that can be of significant benefit for CF patients. 

## 3. Unravelling Nasal/Aerosol Administration of Probiotics in CF

The numerous promising results, but also the relative uncertainty on the use of probiotics in CF, stimulate interest towards the identification of measures to enhance the potential beneficial effects of this kind of intervention. One of these measures, still very poorly investigated in CF, is the administration of probiotics via the respiratory route [46]. Rationally, the lung-targeted administration of probiotics could induce a niche-specific effect directly at the site of infections, possibly resulting in a stronger beneficial outcome than that conferred by the gut–lung axis. Inhaled probiotics could directly compete with pathogens for adhesion to lung mucosa, interfere with pathogen growth and virulence, enhance the integrity of the lung mucosal barrier, and stimulate a local immune response while dampening an excessive inflammatory response (Figure 1). This strategy has demonstrated its efficacy in mice in a number of studies focused on bacterial and viral infections of the respiratory tract [58,59,60,61,62,63,64,65]. Among them, intra-tracheal administration of a mixture of three lactobacilli (*L. fermentum* K.C6.3.1E, *L. paracasei* ES.D.88, *L. zeae* Od.76) was recently demonstrated to protect mice from *P. aeruginosa* pulmonary infection by reducing the bacterial load in the lung and modulating the cytokine levels [64]. These observations highlight that this strategy could be efficacious at least against one of the main pulmonary pathogens in CF. In a subsequent study, the same group screened 50 strains of lactobacilli isolated from CF respiratory samples for their ability to inhibit two of the main *P. aeruginosa* QS-dependent virulence factors (elastase and pyocyanin) [65]. A marked variability in the anti-virulence potential was observed among strains, with 80% of them exhibiting anti-elastolytic activity and 24% anti-pyocyanin activity. Two *Lactobacillus* groups were selected for nasal priming of mice 18 h prior to *P. aeruginosa* PAO1 pulmonary infection. The first group (Lrff), including *L. rhamnosus* and two *L. fermentum* strains, had highly effective anti-PAO1 virulence factor properties, the second one (Lpsb), including *L. paracasei*, *L. salivarius* and *L. brevis*, exhibited no ability to inhibit virulence factor production. Interestingly, both groups of *Lactobacillus* significantly improved 7-day mice survival following challenge with *P. aeruginosa*, suggesting that mechanisms other than the inhibition of virulence may also mediate the protective effect. Additionally, the pulmonary PAO1 burden was significantly reduced at 24 h compared to the control group. Finally, anti-inflammatory activity was observed in mice primed with lactobacilli with a significant reduction in neutrophil recruitment and pro-inflammatory cytokine and chemokine secretion, and an increment in the production of the anti-inflammatory cytokine IL-10 [65].

Not many studies have compared the effects exerted by oral versus respiratory administration of probiotics. In one of these studies, the authors compared the effects of the intragastric and intranasal administration of *Lactobacillus paracasei* NCC2461 on allergic airway inflammation in mice [66]. They found that the application of probiotics directly to the respiratory mucosa was more effective at decreasing markers of allergic reactions (e.g., IL-5, eotaxin) than the intragastric route, possibly due to an increase in regulatory T cells in the lung. 

Airway administration of probiotics is also being explored in humans [67,68,69,70,71,72]. For instance, Marchisio et al. intranasally administered *Streptococcus salivarius* 24SMB, a safe α-haemolytic strain capable of producing bacteriocin-like substances with activity against acute otitis media (AOM) pathogens, to 1–5-year-old children prone to recurrent otitis to evaluate its ability to reduce the risk of new episodes of AOM [67]. Intranasal *S. salivarius* 24SMB or placebo were given twice daily for 5 days each month for 3 consecutive months. The authors evidenced that in the 6 months following the treatment, the number of children who did not experience any AOM or receive antibiotics was lower in the *S. salivarius* 24SMB-treated group than in the placebo-treated group [67]. Such results were confirmed in two subsequent studies administering, via nasal spray, *S. salivarius* 24SMB and *Streptococcus oralis* 89a, two puffs per nostril twice/day for a week for 3 consecutive months [68], or 7 days each month for 5 consecutive months [69], respectively. Importantly, the severity grade of AOM was also significantly reduced after the preventive bacteriotherapy [68].

The efficacy of lactic acid bacterial honeybee microbiota was evaluated in a randomized controlled trial in patients with chronic rhinosinusitis [71]. The patients were asked to self-administer two spray doses to each nostril daily for two weeks, using a nasal spray-device. The honeybee microbiota contained 13 *Lactobacillus* species and 4 *Bifidobacterium* species that are symbionts of *Apis mellifera* and are considered safe for humans. Although well tolerated by the patients, in this case, the bacteriotherapy affected neither symptom severity nor the microbiological flora/local inflammatory activity, suggesting that the choice of probiotic(s) to be used in a specific pathological condition is an essential step to achieving beneficial effects.

It is important to note that although the main direction of the crosstalk in the gut–lung axis seems to occur from the gut to the lung, evidence also exists that the lung microbiota might influence the gut microbiota structure and inflammatory state [31]. This means that the gut–lung axis is bidirectional and that microbial lung changes elicited by administration of probiotics via the respiratory route may have the potential to affect the gut microbiota in CF patients (Figure 2).

Overall, the reported studies suggest that probiotic administration via the respiratory route is a feasible interventional measure that may offer health benefits in specific respiratory disorders. Nevertheless, this measure is still in its infancy and intensive studies to identify the best probiotic/probiotic combinations, intervention schedule and mode of administration for specific airway pathological conditions are needed to fully explore the real therapeutic potential of this innovative intervention. 

## 4. Open Questions for Respiratory Delivery of Probiotics in CF

### 4.1. Choice of the Strain(s) with Activity against CF Pathogens

The growing awareness of the health benefit elicited by probiotics has dramatically increased the variety of products offered on the market over the last few years [73]. Nevertheless, the effects of probiotics are known to be largely species- or strain-specific, demanding for wide screening studies aiding the selection of the best strain or combination of strains to target disease-specific and niche-specific pathogens. For instance, lactobacilli strains, isolated from the oral cavity of healthy volunteers (67 strains) or raw milk (20 strains), were tested for their ability to inhibit biofilm formation and elastolytic activity by *P. aeruginosa*, one of the main CF pathogens [74]. Among them, only five strains (four *L. fermentum* strains and one *L. paracasei* strain), all isolated from the oral cavity, were able to inhibit biofilm formation of *P. aeruginosa* PAO1 in a statistically significant manner. A different group of five strains, not overlapping with the previous one (four *L. fermentum* strains isolated from raw milk and one *L. zeae* strain isolated from the oral cavity), significantly inhibited the elastolytic activity of *P. aeruginosa* PAO1 after 22 h of co-incubation with the bacterium. The lactobacilli strains also differed for their growth ability and acidification properties in artificial saliva [74]. As reported above, high degree of variability in the antivirulence potential against *P. aeruginosa* was also reported among *Lactobacillus* strains isolated from CF respiratory samples [65]. In another study, 57 *Lactobacillus* strains isolated from milk and yogurt samples were tested for their ability to inhibit the growth and biofilm formation of 80 *P. aeruginosa* strains [75]. The screening found two strains, identified as *L. fermentum* by biochemical and 16S rDNA techniques, showing a broad inhibition/killing effect and anti-biofilm effect on all the *P. aeruginosa* strains. Investigation of the mechanism of the inhibitory effect revealed that it was not due to bacteriocin production, but rather to the production of three main organic acids including lactic acid, acetic acid and formic acid. As medium acidification is considered one of the main probiotic-mediated antibacterial mechanisms, and *P. aeruginosa* is quite sensitive to low pH, the ability of lactobacilli to grow and promote the acidification of the CF lung-like environment might be an important criterion for the selection of probiotics to administer to CF patients via the respiratory route. 

Inhibitory effects of different probiotic strains were also reported against another relevant CF lung pathogen, *Staphylococcus aureus* [76,77]. The effects were mediated both by direct competition as well as by production of acids or bacteriocin-like inhibitors. *L. acidophilus* also inhibited *S. aureus* biofilm formation and lipase production. In vitro antimicrobial activity did not necessarily correlate with in vivo efficacy in animal infectious models highlighting that in vivo testing in mice or in less ethically demanding models (e.g., *Galleria mellonella*) might be a necessary step in the screening of probiotics before proceeding to clinical studies. To the best of our knowledge, effects of probiotics against *S. aureus* strains specifically isolated from the lungs of CF patients alone or co-cultured with CF *P. aeruginosa* strains is still a highly unexplored area of research. 

### 4.2. Lung Colonization Capability

It is known that the lung environment in CF displays specific features as a direct consequence of the CTFR dysfunction including the accumulation of a dense mucus, hypersecretion of mucins, neutrophilic infiltration, altered pH of the airway surface liquid, pro-inflammatory milieu, high extracellular DNA content and low oxygen tension [78,79]. This raises the question of whether the lung colonization capability of probiotic strains can be effective in such an altered environment. Again, specific selection criteria are needed in order to identify the right probiotic(s) harboring the appropriate features to colonize the CF-lung ecological niche. Most of the commercial strains are intended for oral administration, and therefore, are selected for their ability to pass the gastric barrier and adhere to intestinal cells. These same properties do not necessarily coincide with those required to settle and survive in the lung. In this regard, it might be argued that strains isolated from CF lung, presumably already adapted to such a harsh environment and able to compete with the indigenous microflora, can provide a valuable source of lactobacilli. Indeed, the high prevalence of lactobacilli carriage has been found in CF patients with 61% of them harboring at least one strain of *Lactobacillus* in their sputum, with *L. rhamnosus*, *L. fermentum*, *L. paracasei* and *L. gasseri* being the four most common species [80]. An eight-month follow-up of 26 patients revealed that in the majority of them (20/26), the colonization was transient, while 23% of them (6/26) were chronically colonized [80], suggesting a high degree of adaptation to the local conditions of the respective strains. 

### 4.3. Mode of Administration and Formulation

A key issue for probiotic lung delivery is the mode of administration and formulation. As probiotics are living microorganisms, they might be easily inactivated during preparation, storage or administration with a consequent reduction in their therapeutic potential. Various approaches relying on nanotechnology and microencapsulation have been reported as valid means for stabilizing and protecting probiotics from adverse conditions by providing them a physical barrier that enhances stability, delivery and functionality, although encapsulation itself may negatively affect viability [81]. 

In addition, each method of administration has its own limitations. For instance, potential factors that may hamper the airway delivery of probiotics are rapid mucociliary clearance, short retention time, enzymatic degradation, low stability in mucus and competition with lung indigenous or pathogenic microflora. Nasal delivery of probiotics via nasal spray seems a promising and feasible administration approach that has demonstrated its efficacy in animal models and clinical studies [82], but other ways of delivery (e.g., aerosol) that ensure a high volume and efficient arrival of therapeutic bacteria to the lower respiratory tract are worth being explored. 

Modern formulation technologies and biomaterials are being intensively investigated and hold promise for providing optimized production processes and sophisticated delivery systems with the controlled release of probiotics for site-specific applications in the years to come [81,82]. Specifically, some nanomaterial-based formulations including nanofibers, nanoparticles and nanocomposites seem to meet most of the requirements for delivery systems of probiotics including efficient encapsulation, controlled release and improved stability of the therapeutic bacteria during manufacturing, storage and in vivo applications [82]. For instance, in a recent study, Jokicevic and coworkers analyzed several spray-dried formulations for probiotic nasal delivery [83]. They screened different combinations of excipients (sugars, sugar-alcohols and polymers) for their ability to preserve the viability of a strain of *L. casei* after spray drying and during a period of 7 months of shelf-life. The best formulation yielded approximately 3 × 10^10^ CFU/gr after storage, with only a 0.3–0.4 log reduction as compared to the initial load. Resuspension of the powder and dispersion through a nasal spray bottle did not lead to significant viability loss. Finally, functional tests demonstrated that some of the formulations maintained a high capability of adhering to the human airway epithelial cell line Calu-3 as compared to fresh *L. casei* cells and good antimicrobial activity against common upper respiratory tract pathogens [83]. The study highlights that the final benefits supplied by probiotics might be not only strain-specific but also formulation- and process-dependent.

### 4.4. Timing of the Intervention 

Another open question on the airway delivery of probiotics to CF patients regards the timing of intervention also in relation to other treatments. In order to obtain the best beneficial outcome, should probiotics be administered to CF patients as a prophylactic strategy to prevent the insurgence of pulmonary exacerbations or, rather, as a therapeutic intervention during exacerbations, as adjunctive therapy to antibiotics? Both approaches seem rational. The preventive approach may provide a microbiological barrier to hinder lung colonization by pathogenic bacteria through exclusion or competition mechanisms (Figure 1). In line with this view, priming with intranasal administration of lactobacilli has been demonstrated to prevent *P. aeruginosa* acute pneumonia in mice [65], while preventive treatments with *S. salivarius* and *S. oralis* have proved its efficacy in reducing recurrent otitis media in children [67,68,69]. On the other hand, a therapeutic approach carried out during or after antibiotic therapy could take advantage of the antibiotic-mediated reduction in the pathogenic load and consequent increase in the availability of adhesion sites in the lung while contributing to the repopulation of the pulmonary ecosystem with beneficial bacteria. Evidence of the increased lung colonization rate of *L. fermentum* given intranasally to mice simultaneously with ampicillin administration via the oral route have been previously reported [84]. 

The best administration schedules (daily, weekly, monthly) in preventive and/or treatment interventions, the doses and mode of administration for efficacious pulmonary delivery, as well as possible interference with other CF-specific treatments (e.g., CTFR-modulators, antibiotics) are all variables that will need rigorous evaluation in vitro, ex vivo and in vivo studies.

## 5. Conclusions

The reduced availability of efficacious antibiotics due to the rapid spread of bacterial resistance world-wide has given new impetus to the identification of new strategies to control infections. Bacteriotherapy, i.e., the use of beneficial bacteria to prevent/treat infections, is gaining increasing interest outside its traditional gastrointestinal tract application. Specifically, evidence is accumulating that administration of probiotics via the oral route can produce beneficial effects in organs located distantly to the gut, including the respiratory tract. In CF, oral administration of probiotics has proven to partially restore gut dysbiosis, reduce intestinal inflammation, and lower lung infections and exacerbation rate in some, but not all the studies, providing opportunities, but also challenges for a routine implementation of such an intervention.

**Figure 3:**
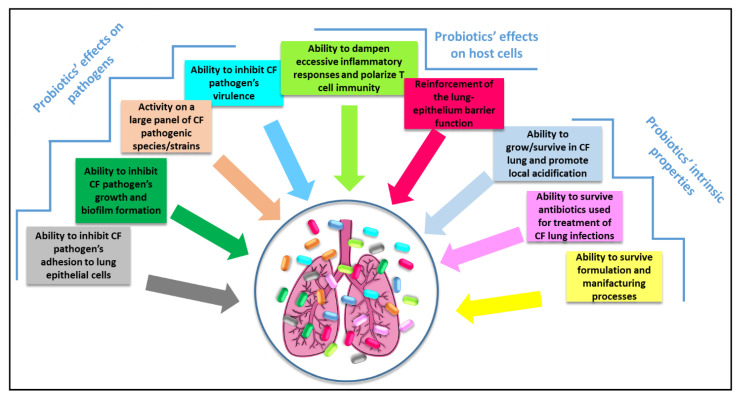
Ideal requirements of a probiotic/probiotic mix for administration to CF patients via the respiratory route that can guide the selection process.

On the basis of the published literature, administration of probiotics via the respiratory route seems feasible and likely to provide improved beneficial outcomes by virtue of its direct effect on the lung ecosystem. Nevertheless, much work still needs to be done to identify the ideal lung-targeted probiotic or probiotic mix able to contrast CF-specific pathogens, survive and colonize the CF-lung, modulate host responses, and survive formulation and manufacturing processes (Figure 3). In addition, evidence-based probiotic selection has to be evaluated in adequately powered multicenter clinical studies in order to identify the dose, mode and rhythm of administration to yield the desired beneficial effects. Although the advent of triple CTFR modulator therapy has profoundly changed the CF panorama significantly improving the life quality of CF patients, the effects of these new therapeutical approaches on lung infections have yet to be determined. In addition, a significant proportion of CF patients (up to 20%) are not eligible or not responsive to such treatments [85], highlighting the importance of pursuing the search for new and effective therapies for the management of lung infections in CF patients.

## Figures and Tables

**Table 1:** Opportunities and challenges of the oral administration of probiotics in CF.

	Strain(s) Used	Administration Schedule	Reported Effects/ Main Conclusions	References
**Opportunities**	Different probiotic species/strains in different studies	Different dose/treatment regimens	no or minimal adverse events (mild flatulence, vomiting, diarrhea and allergic reactions)	[14,16]
	*Lactobacillus rhamnosus* GG (LGG)	5 × 10^9^ CFU/day for 1 month	Reduction in fecal calprotectin concentration	[17]
	*Lactobacillus rhamnosus* GG (LGG)	6 × 10^9^ CFU/day for 1 month	Amelioration of intestinal dysbiosis (increase in *Bacteroidetes* counts) Reduction in fecal calprotectin concentration	[18]
	*Lactobacillus casei, Lactobacillus rhamnosus, Streptococcus thermophilus, Bifidobacterium breve, Lactobacillus acidophilus, Bifidobacterium infantis, Lactobacillus bulgaricus*	1 × 10^9^ CFU/day for 1 month	Reduction in fecal calprotectin concentration	[19]
	Synbiotic preparation (fructooligosaccharides + *L. paracasei, L. rhamnosus, L. acidophilus, and B. lactis*)	90-day of supplementation	Significant reduction in serum IL-6 and IL-8 in CF patients with positive bacteriology	[20]
	*Lactobacillus reuteri* DSM 17938	10^8^ CFU in a chewable tablet (one tablet per day, for 6 months)	Reduction in the γ-*Proteobacteria* phylum; increment in microbial diversity with a higher representation of *Firmicutes*; overall improvement of the gastrointestinal comfort evaluated via the Gastrointestinal Quality of Life Index (GIQLI)	[21]
	*Lactobacillus acidophilus, Lactobacillus bulgaricus, Bifidobacterium bifidum, Streptococcus thermophiles*	6 × 10^9^ CFU/day for 6 months	Reduction in pulmonary exacerbations in comparison to the previous 2 years and to 6 months post-treatment	[22]
	*Lactobacillus rhamnosus GG (LGG)*	6 × 10^9^ CFU/day for 6 months	Reduction in pulmonary exacerbations and hospital admissions	[23]
**Challenges**	*Lactobacillus rhamnosus GG (LGG)*	6 × 10^9^ CFU/day for 6 months	No effect on the number of pulmonary exacerbations, number of hospitalizations, days in therapy and number of episodes treated with oral therapy	[24]
	*Lactobacillus rhamnosus SP1 (DSM 21690) and Bifidobacterium animalis spp.BLC1 (LGM23512)*	10^10^ CFU/day for 4 months	No effect on fecal calprotectin, pulmonary function, pulmonary exacerbations and microbiome	[25]
	Single strains or multi-strain combination in different studies	Doses ranging from 10^8^ to 10^10^	Limited evidence to support probiotic use due to lack of well-designed and adequately powered trials	[14,26,27,28]
	*Lactobacillus casei, Lactobacillus rhamnosus, Streptococcus thermophilus, Bifido-bacterium breve, Lactobacillus acidophilus, Bifidobacterium infantis, and Lactobacillus bulgaricus*	2 × 10^9^ CFU; 2 capsule per day for 1 month	Effects of probiotics seem to be temporary	[29]
	-	-	Lack of a rational selection of probiotic strains specifically targeting CF pathogens	This article
